# Silica Nanoparticle–Induced Reproductive Toxicity in Male Albino Rats via Testicular Apoptosis and Oxidative Stress

**DOI:** 10.1007/s12011-022-03280-w

**Published:** 2022-06-07

**Authors:** Rehab A. Azouz, Reda M. S. Korany, Peter A. Noshy

**Affiliations:** 1 Toxicology and Forensic Medicine Department, Faculty of Veterinary Medicine, Cairo University, Giza, 12211 Egypt; 2 Pathology Department, Faculty of Veterinary Medicine, Cairo University, Giza, 12211 Egypt

**Keywords:** Silica nanoparticles, Testes, Histopathology, Caspase-3, iNOS

## Abstract

Amorphous silica nanoparticles (SiNPs) are being utilized in different fields such as medicine, cosmetics, and foods. However, the causes and mechanisms underlying SiNP testicular damage remain largely unclear. In the present study, we aimed to investigate this issue. Thirty male rats were randomly divided into three groups: control group (*n* = 10), 500 ppm SiNP–treated group (*n* = 10), and 1000 ppm SiNP–treated group (*n* = 10). SiNPs were given orally in drinking water for 30 days. Micronucleus assay was performed on blood RBCs. The concentrations of testicular malondialdehyde (MDA) and glutathione (GSH) and catalase (CAT) activity were measured. Moreover, the histopathological alterations and the expression of apoptotic (caspase-3) and pro-inflammatory and oxidative stress markers (iNOS) in testes and epididymis were analyzed and compared between the three groups. The results showed an increased level of micronucleus frequencies in the 1000 ppm–treated group, as well as increased levels of MDA and decreased activity of CAT and GSH content in testicular tissues in the 1000 ppm–treated group, suggesting DNA damage and oxidative stress mechanisms. Also, there were significant testicular histopathological alterations in this group. Furthermore, 1000-ppm SiNPs could enhance testicular apoptosis, inflammation, and oxidative stress by increasing the expression of apoptotic, pro-inflammatory, and oxidative stress genes including caspase 3 and iNOS in the examined tissue. The lower concentration of SiNPs did not produce any significant biochemical, histopathological, or immunohistochemical alterations whereas 1000-ppm SiNPs resulted in significant testicular changes by exacerbating apoptotic, inflammatory, and oxidative stress–mediated testicular damage.

## Introduction

Nanotechnology is the plan, portrayal creation, and utilization of structures, gadgets, and frameworks by controlling the shape and size at the nanometer scale [1]. Recently, different types of nanomaterials have been designed and produced. The small particle size and large surface area make the nanomaterials able to display several useful properties that are different from those of other bulk materials [2]. Amorphous silica nanoparticle has a variety of unique properties, as ease of synthesis, relatively low cost, and availability of sites for surface modifications [3]. The amorphous silica nanoparticle is being synthesized with highly tunable biocompatibility and stability, which is considered to be very promising for many applications, as drug delivery and molecular imaging. Heavy production and excessive use of silica nanoparticle have resulted in an increased risk of human exposure [4]. Previous studies suggested that nanoparticles might exert a toxic effect on the human reproductive system [5]. The unique nature of the male reproductive system makes the biological effect of nanoparticles on this system increasingly found as an important part of the overall toxicological studies [4]. Recent studies have found that 60-nm SiNPs can be deposited in the mitochondria of spermatogenic cells and decrease sperm quality followed by other abnormalities [6]. Amorphous silica nanoparticles could cross the cytomembrane, enter spermatocyte, and induce apoptosis by targeting microRNA death receptors. Moreover, other previous studies have found that amorphous SiNPs induce male reproductive toxicity by causing abnormal mitosis, which is mediated by the PKC-δ pathway in spermatocyte, resulting in the inhibition of the initiation and progression of meiosis [7]. Previous studies found that silica nanoparticles influenced the maturation of sperm in the epididymis, decreased the quantity and quality of epididymis sperm, and led to energy metabolism dysfunction resulting from damage of the mitochondrial structure. However, the specific pathological mechanism of SiNP-induced dysfunction of the male reproductive system is still unclear [5].

## Aim of Work

As silica nanoparticles are widely used in many industries as cosmetics, foods, and drugs, it is important to ensure the reproductive safety of these nanoparticles and investigate their biological effects at different concentrations.

## Materials and Methods

### Silica Nanoparticles

Silica nanoparticles (99.5% trace metal basis, 5–20 nm particle size in TEM) were purchased from Sigma-Aldrich Chemical Co. (St. Louis, MO, USA). Before use, the SiNPs were autoclaved for 2 h (h), vortexed for 1 min, and then sonicated for 5 min (120 W output; Thermo Scientific, USA).

### Experimental Design

Thirty Sprague Dawley male rats (180–200 g) of 8 weeks of age were obtained from the Faculty of Veterinary Medicine – University of Sadat City. Rats had ad libitum access to basal basic diet (Table 1) and tap water. All rats were acclimatized for 2 weeks before the beginning of the experiment. Animal handling and treatment procedures were conducted according to the Guidelines for the Care and Use of Laboratory Animals of the National Institutes of Health (NIH) and approved by a research ethics committee of the Faculty of Veterinary Medicine—Cairo University (VetCU1022019079). Rats were randomly divided into three equal groups (*n* = 10). Group I (Control) received distilled water, group II received 500-ppm silica nanoparticles in drinking water, and group III received 1000-ppm silica nanoparticles in drinking water for 30 days according to the method described by [8].

**Table 1:** Diet formulation

Ingredients	Normal basic diet (gm)
Casein	12
Safflower oil	10
Sucrose	23
Starch	45.5
Vitamin mix	1
Mineral mix	3.5
Cellulose	5

### Sampling

At the end of the designed period (30 days), rats were anesthetized, the blood samples were collected from median canthus (orbital vessels). Blood was collected in heparinized tubes and immediately used for micronucleus assessment. Then, rats were euthanized by cervical dislocation, Tissue samples from testes were also collected, and part of them was homogenized in ice-cold 100 mM phosphate buffer (pH 7.4) using a Teflon homogenizer. The homogenates were centrifuged at 14,000 × g for 20 min, and the resulting supernatant was kept in a deep freezer at − 20 °C for antioxidant parameters assay. Another part of the testes and epididymis was collected and fixed in 10% buffered neutral formalin solution for histopathological and immunohistochemical examinations.

### Micronucleus Assay

Drops of whole blood were immediately smeared on glass slides. The slides were air-dried for 24 h, fixed in methanol for 10 min, then stained with 10% Giemsa stain for detection of micronuclei in erythrocytes; a × 1000·oil-immersion lens was used to examine the slides. The mean frequency of micronuclei was evaluated in 1000 cells per group [9].

### Assessment of Oxidative Stress

Malondialdehyde (MDA) level (CAT. No. MD 25 29), catalase (CAT) activity (CAT. No. CA 25 17), and reduced glutathione (GSH) content (CAT. No. GR 25 11) were estimated in testicular tissue homogenate using kits obtained from Biodiagnostic Co., Giza, Egypt.

## Malondialdehyde Level Estimation

Lipid peroxidation was determined indirectly by measuring the production of malondialdehyde (MDA) in the testis extract according to the method of [10] which was based on TBA reactivity. The reaction between lipid peroxide and thiobarbituric acid in an acidic medium to produce a reactive colored product was estimated. The color intensity of this product is directly proportional to the MDA content. Absorbance was measured at 534 nm.

## Catalase Activity Assessment

Catalase (CAT) activity was measured by the UV colorimetric method of [11] using H_2_O_2_ as substrate. The remaining H_2_O_2_ reacts with 3,5-dichloro-2-hydroxybenzene sulfonic acid and 4-aminophenazone in the presence of peroxidases to produce a chromophore. The color intensity of the chromophore is inversely proportional to CAT activity. Absorbance was measured at 510 nm.

## Reduced Glutathione Content Detection

Reduced glutathione (GSH) level was measured colorimetrically according to the method of [12] as the reduction of 5,5-dithiosbis-2-nitrobenzoic acid with GSH producing a yellow substance. The intensity of the color is directly proportional to the GSH content. Absorbance was measured at 405 nm.

### Histopathological Examination

Tissue specimens from testes and epididymis were collected from experimental groups at the end of the experiment, fixed in neutral buffered formalin 10%, washed, dehydrated, cleared, and embedded in paraffin. The paraffin-embedded blocks were sectioned at 5-micron thickness and stained with hematoxylin and eosin [13] for histopathological examination. Stained sections were examined by a light microscope (Olympus BX50, Japan).

### Histopathological Lesion Scoring

Histopathological alterations in testes and epididymis were recorded and scored as no changes (0), mild (1), moderate (2), and severe (3) changes; the grading was determined by percentage as follows: < 30% changes (mild change), < 30–50% (moderate change), and > 50% (severe change) [14].

### Immunohistochemistry

Immunohistochemical analysis was carried out following the methods described by [15]. The tissue sections from testes and epididymis were deparaffinized in xylene and rehydrated in different grades of alcohol. The antigen retrieval was done by pretreating the sections with citrate buffer of pH 6 for 20 min. Sections were incubated with rabbit polyclonal anti-caspase-3 antibody at a concentration of 1:1000 (ab4051; Abcam, Cambridge, UK) and rabbit polyclonal anti-iNOS antibody at a concentration of 1:100 (ab15323; Abcam, Cambridge, UK) for 2 h in a humidified chamber. The sections were incubated with goat anti-rabbit IgG H&L (HRP) (ab205718; Abcam, Cambridge, UK), and 3,3′-diaminobenzidine tetrahydrochloride (DAB, Sigma) was used as a chromogen. Finally, the slides were counterstained with hematoxylin and mounted with DPX. The negative control slides were prepared by replacing primary antibodies using PBS.

### Evaluation of Caspase-3 and iNOS Immunostaining

The quantitative immunoreactivity of caspase-3 and iNOS was evaluated in tissue sections in each group [16], and five tissue sections were examined. Immuno-reactivity was analyzed in 10 microscopical fields per section under a high-power microscopic field (× 400). The percentage of positively stained cells (%) was estimated by color deconvolution ImageJ 1.52 p software (Wayne Rasband, National Institutes of Health (USA)).

### Statistical Analysis

The SPSS computer program was used for statistical analysis. A statistical analysis was carried out by one-way ANOVA setting the probability level to *p* < 0.05; a post hoc analysis of group differences was performed by LSD test. Data was expressed as mean ± SEM.

## Results

### Micronucleus Assay

Micronuclei (MN) in rat erythrocytes were assessed in all treated groups (Table 2). Micronucleus frequencies per 1000 erythrocytes were significantly higher in the 1000 ppm SiNP–treated group (0.6) than the control (0.13) and 500-ppm SiNP groups (0.14) (*p* ≤ 0.05).

**Table 2:** Micronucleus frequency (MN/1000 erythrocytes) of all treated groups

Control	500 ppm	1000 ppm
0.13 ± 0.03	0.14 ± 0.05^b^	0.6 ± 0.03 ^a^

Values are presented as mean ± SE (*n* = 10 rats/group)

^a^Presence of a statistically significant difference from the control group

^b^Presence of a statistically significant difference from the 1000-ppm SiNP group at *p* < 0.05

### Oxidative Stress Assessment Findings

Oxidative stress is one of the potential mechanisms of reproductive toxicity. As shown in Table 3, MDA level (level of lipid peroxidation) was increased in 1000 ppm SiNP–treated rats. The activity of CAT and the content of GSH of the testicular tissues showed a significant decrease in 1000 ppm SiNP–exposed animals in comparison to the 500 ppm SiNP–treated group and the control one.

**Table 3:** Effect of SiNP at the testicular MDA level, CAT activity, and GSH content in different studied groups

	Control	500 ppm	1000 ppm
MDA (nmol/g)	95.6 ± 3.7	96.1 ± 4.5^b^	119.1 ± 1.6^a^
Catalase (U/g)	138.5 ± 5.8	137.4 ± 4.7^b^	123.7 ± 2.4^a^
GSH (mg/g)	92.6 ± 1.4	92.4 ± 2.6^b^	78.3 ± 4.4^a^

Values are presented as mean ± SE (*n* = 10 rats/group)

^a^Presence of a statistically significant difference from the control group

^b^Presence of a statistically significant difference from the 1000-ppm SiNP group at *p* < 0.05

### Histopathological Findings

**Fig. 1:**
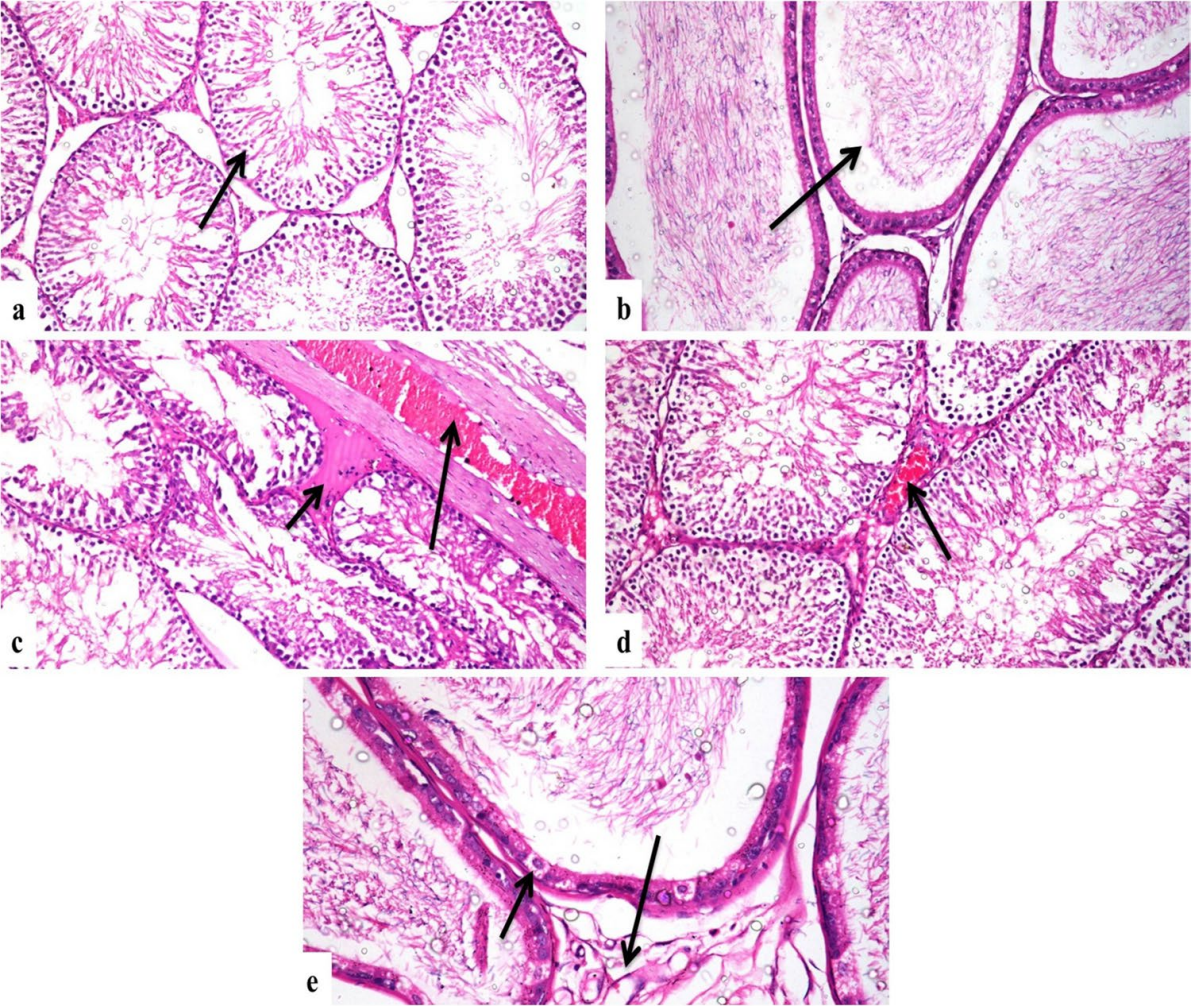
Photomicrograph, rat **a** testes of the control group, showing a normal histological structure of seminiferous tubules (arrow) (H&E × 200), **b** epididymis of the control group, showing a normal histological structure of epididymal tubules (arrow) (H&E × 200), **c** testes of the 500 ppm–treated group, showing congestion and thickening of tunica albuginea (long arrow) and interstitial edema (short arrow) (H&E × 200), **d** testes of the 500 ppm–treated group, showing mild congestion of interstitial blood vessels (arrow) (H&E × 200), **e** epididymis of the 500 ppm–treated group, showing mild vacuolar degeneration of tubular lining epithelium (short arrow) and interstitial edema (long arrow) (H&E × 400)

**Fig. 2:**
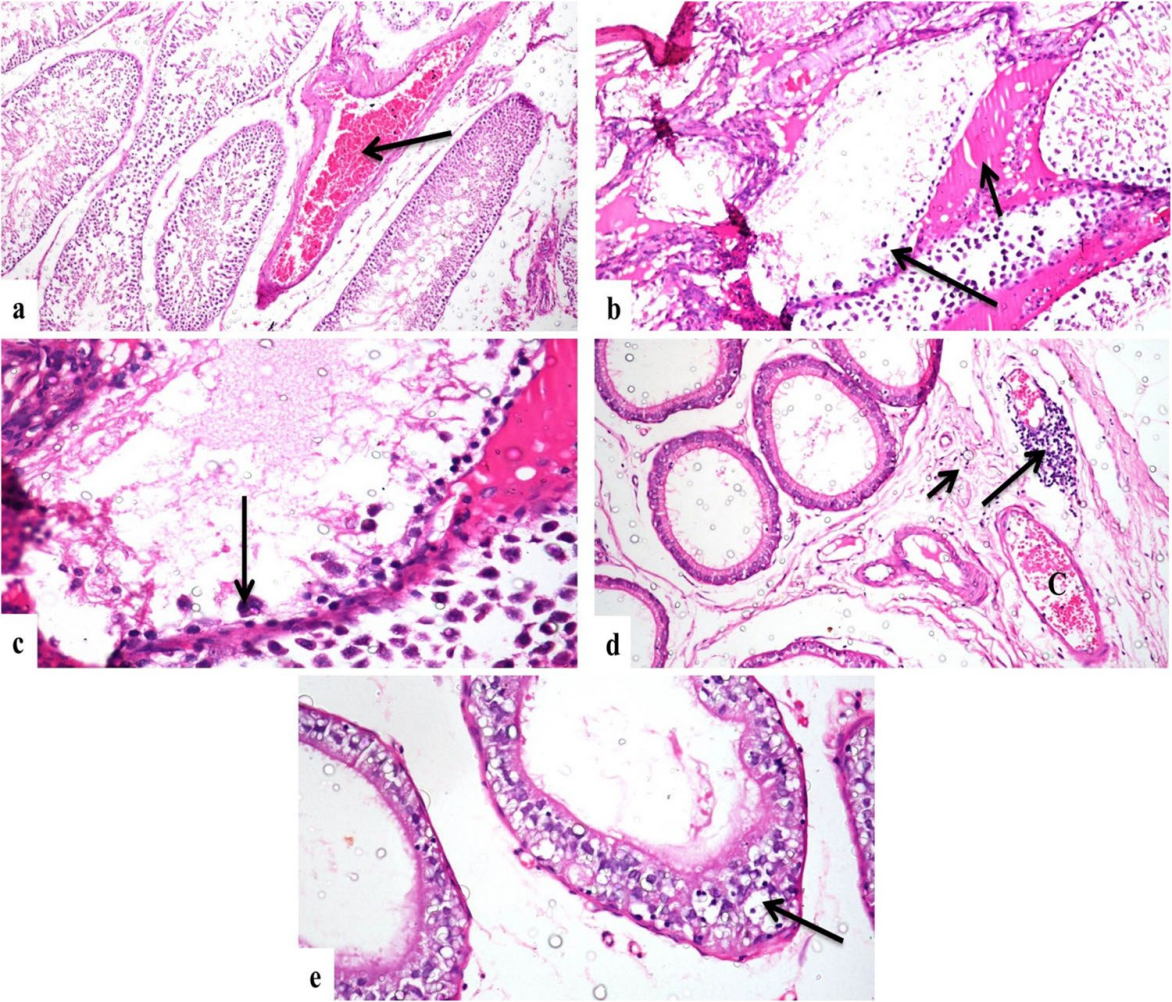
Photomicrograph, 1000 ppm–treated group **a** testes showing severe congestion of interstitial blood vessels (arrow) (H&E × 200), **b** testes showing severe degeneration of seminiferous tubules (long arrow) and interstitial edema (short arrow) (H&E × 200), **c** higher magnification of the previous photo showing seminiferous tubules devoid of spermatogenic cells with presence of spermatid giant cells (arrow) (H&E × 400), **d** epididymis showing interstitial edema (short arrow), perivascular lymphocytic cuff (long arrow), and vascular congestion (C) (H&E × 200), **e** epididymis showing severe vacuolar degeneration and necrosis of tubular lining epithelium (arrow) (H&E × 400)

Testes of the control group showed a normal histological structure of seminiferous tubules, interstitial tissue, and tunica albuginea (Fig. 1a). Concerning the 500 ppm nanosilica–treated group, it revealed mild congestion of interstitial and tunica albuginea blood vessels with mild interstitial edema, and there was a mild testicular degeneration in a few seminiferous tubules (Fig. 1c and d). The 1000 ppm nanosilica–treated group revealed severe congestion of interstitial and tunica albuginea blood vessels (Fig. 2a), severe testicular degeneration in form of few spermatogenic cells lining the seminiferous tubules, and presence of spermatid giant cells; cystic dilatation of some seminiferous tubules was evident; and there was also interstitial edema (Fig. 2b and c). Epididymis of the control group showed a normal histological structure (Fig. 1b). The 500 ppm nanosilica–treated group showed vacuolar degeneration in few epithelial cells lining epididymal tubules with mild interstitial edema and congestion (Fig. 1e). The group intoxicated with 1000 ppm nanosilica showed interstitial edema and congestion of interstitial blood vessels with infiltration of mononuclear inflammatory cells (Fig. 2d). There were necrosis and degeneration of the most epididymal tubular lining epithelium which appeared devoid of sperms (Fig. 2e).

### Histopathological Lesion Score

All recorded lesions in testes and epididymis were scored according to their severity as shown in Table 4.

**Table 4:** Scoring of histopathological alterations in testes and epididymis of experimental groups

Lesions	Control	500 ppm	1000 ppm
-Testicular degeneration	0	1	3
-Edema of interstitial tissue of testes	0	1	2
-Congestion of interstitial blood vessels of testes	0	1	3
-Thickening and congestion of tunica albuginea	0	1	2
-Degeneration of epididymal tubules	0	1	3
-Interstitial edema and congestion of epididymis	0	1	3
-Infiltration of mononuclear cells in interstitial tissue of epididymis	0	0	1

The score system was designed as: score 0 = absence of the lesion in all rats of the group (*n* = 5), score 1 =  < 30%, score 2 =  < 30–50%, score 3 =  > 50%

### Immunohistochemical Findings for Caspase-3 and iNOS Expression

**Fig. 3:**
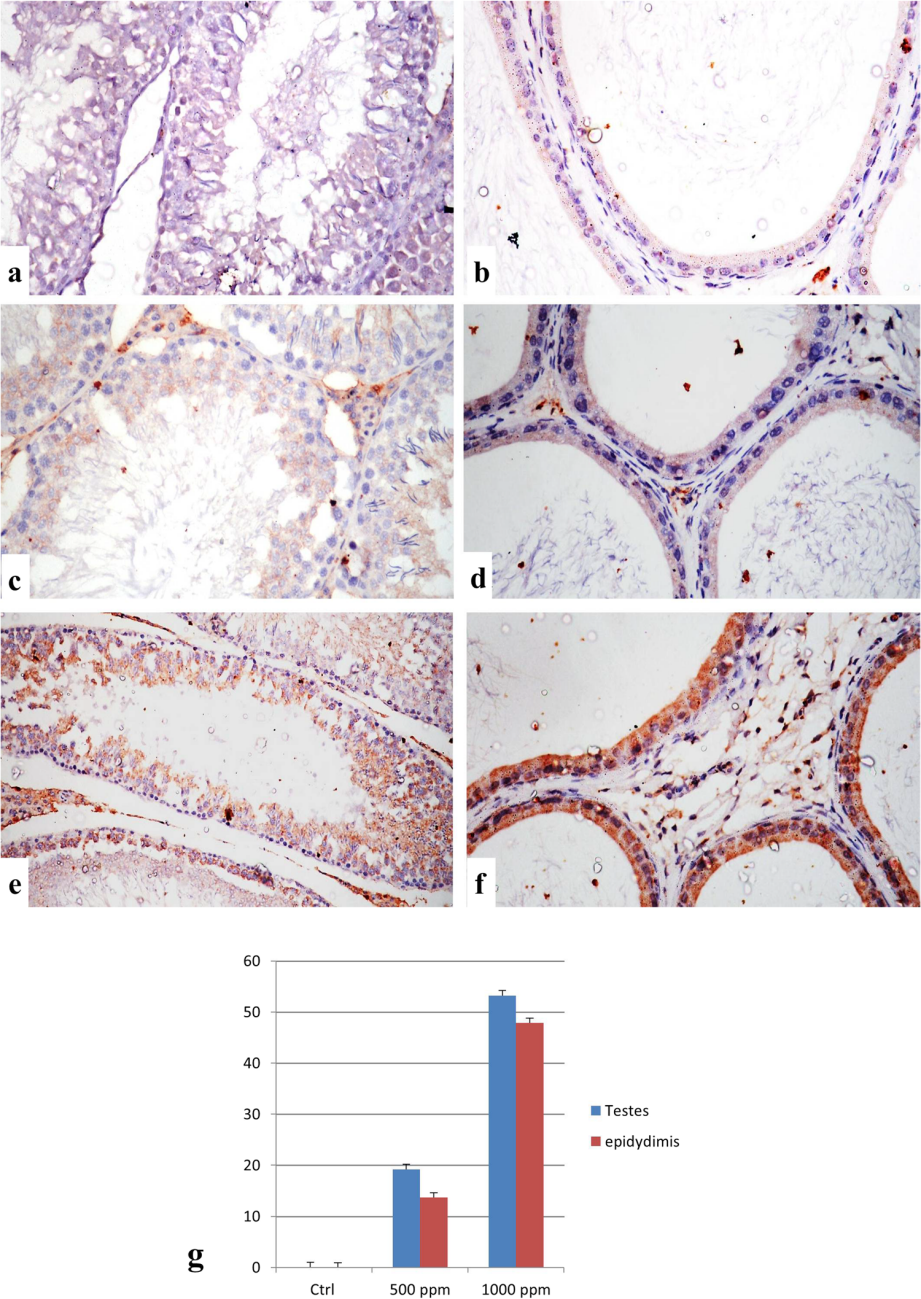
Immunostaining of caspase-3 in testes and epididymis. **a**, **b** Control group showing no immune reactive cells. **c**, **d** The 500 ppm–treated group showing a weak positive expression of caspase-3. **e**, **f** The 1000 ppm–treated group showing a strong positive expression of caspase-3 (caspase-3 × 400). **g** Immuno-staining expression of caspase-3% area in testes and epididymis. Data was expressed as mean ± SEM (*n* = 5)

**Fig. 4:**
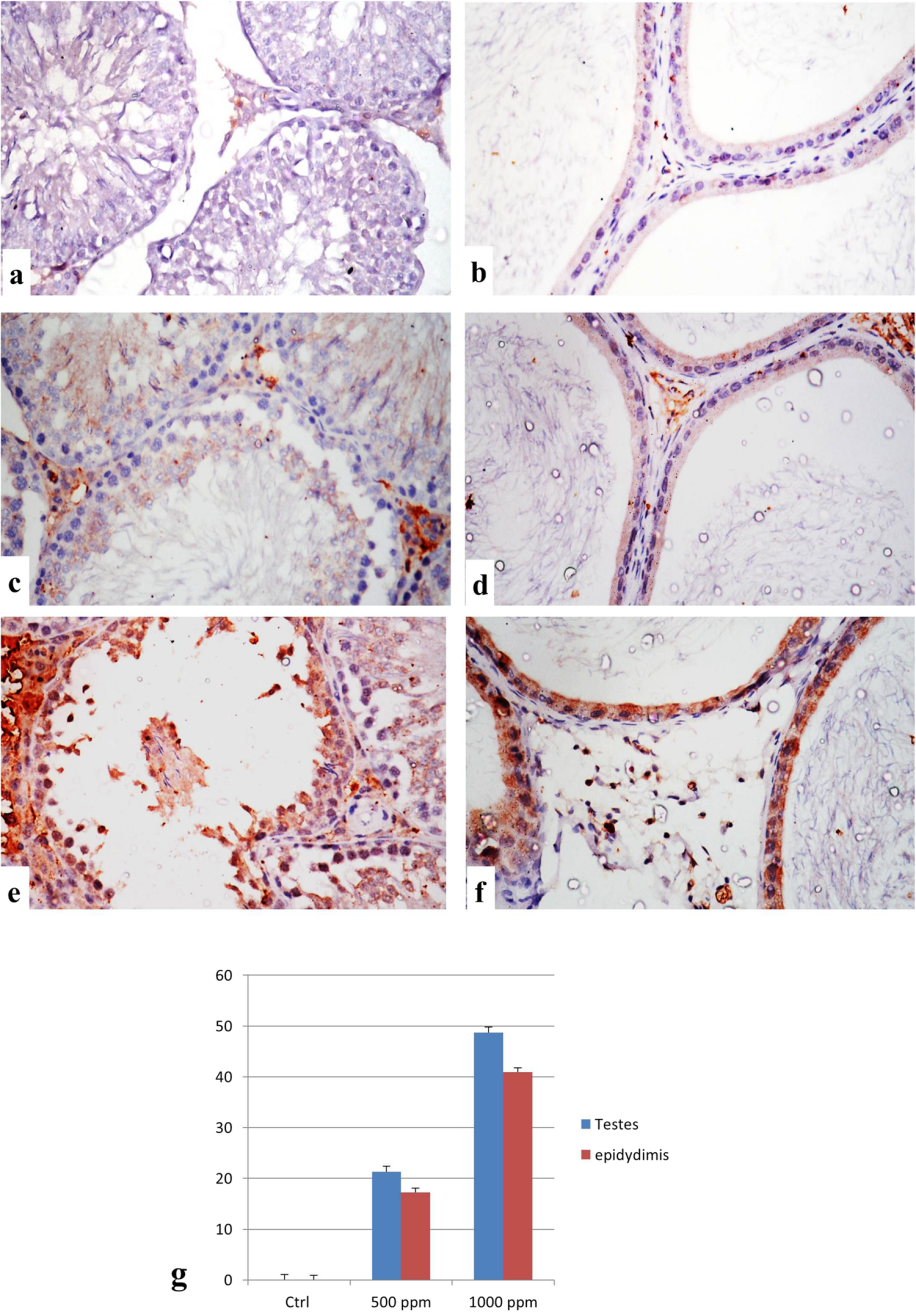
Immunostaining of iNOS in testes and epididymis. **a**, **b** Control group showing no immune reactive cells. **c**, **d** The 500 ppm–treated group showing a weak positive expression of iNOS. **e**, **f** The 1000 ppm–treated group showing a strong positive expression of iNOS (iNOS × 400). **g** Immuno-staining expression of iNOS % area in testes and epididymis. Data was expressed as mean ± SEM (*n* = 5)

The immunostaining expression of caspase-3 and iNOS % area in the testes and epididymis of different experimental groups is shown in Figs. 3g and 4g; in the group treated with 1000 ppm nanosilica, the expression % area was significantly higher than the 500 ppm nanosilica–treated group. Control groups showed no immune-reactive cells for both markers (Figs. 3 and 4a and b). Immunostaining of caspase-3 and iNOS in these organs of the 500 ppm nanosilica–treated group revealed a weak expression of both markers (Figs. 3 and 4c and d). The groups treated with 1000 ppm nanosilica showed a strong positive immunostaining expression in the testes and epididymis (Figs. 3 and 4e and f).

## Discussion

As nanomaterials have the potential to enhance the quality of human life, it is important to ensure their safety. Therefore, to know the reproductive safety of SiNPs, it is important to investigate their biological effects on the testis. The testis is a sensitive organ to many toxicants, such as pesticides [17]. Male infertility has been a difficult disorder to solve and caused by dysfunction of the testes [18]. The toxicity of nanomaterials to male reproductive functions has been studied [19]. This study was aimed to study the possible reproductive toxic effect of silica nanoparticle on male albino rats. For this purpose, thirty male albino rats were used and divided into three equal groups (control and 500 ppm and 1000 ppm nanosilica–treated groups). Micronucleus assay, oxidative stress parameters, histopathology, and immunohistochemistry were assessed. MN is considered to be caused by DNA damage or genomic instability [20]; in our study, MN frequencies were significantly higher in the 1000 ppm SiNP–treated group than in the control and 500 ppm SiNP groups. Mn is important for future use as one of the most reliable, well-established, and feasible genotoxicity parameters [21]. The result of the current study has revealed a significant increase in the MDA level (a lipid peroxidation marker), while it recorded a significant decrease in the CAT activity (an antioxidant enzyme) and GSH amount (an antioxidant marker) of 1000 SiNP–exposed animals as compared to the control and 500 ppm SiNP–treated groups. Recently, there has been growing concern about the toxic effects of chemicals on the male reproductive system. The testicular tissue remains attractive to oxidative stress due to the presence of many highly unsaturated fatty acids and an effective reactive oxygen species–producing system [22]. Under normal testicular function, there is a balance between ROS production and antioxidant elimination [23]; when this balance is broken, several cellular components could be injured. Conversely, increased levels of ROS can induce testicular function impairment and oxidative damage. To prevent this, the testis is supplied naturally with a very potent antioxidant system that protects it from the damaging effects of free radicals. The glutathione, superoxide dismutase, catalase, and several non-enzymatic antioxidants all protect the testes by counteracting any oxidative stress [22]. However, overexposure to environmental pollutants has been shown to impair the pro-oxidant/antioxidant equilibrium in the testes and thereby affect testicular function [24]. Histopathologicaly, with a high concentration of silica nanoparticles, the testes showed severe testicular degeneration and this change comes in accordance with [4]. Also, the epididymis showed vacuolar degeneration, necrosis, edema, and lack of sperms in epididymal tubules; these results were compatible with that recorded by [7]. Silica nanoparticles might cause damage to the histological structure of testes, block spermatogenesis, and decrease the quantity and quality of sperm [4]. Silica nanoparticles cause testicular damage by reducing the ATP level and affecting expressions of regulatory factors of meiosis. SiNP has also been found to pass through the blood-testis barrier and nuclear membranes of spermatocytes. Epididymis provides a place for sperm maturation. The disorganized epididymal tubular lining epithelium suggested the destruction of the blood-epididymis barrier, which results in the stagnation of sperm maturation and post-testicular male infertility. So, nanoparticles might pass through the barrier and deposit in the epididymis, causing an irregular cell arrangement and deformed epididymal tubules [25]. In our study, it was found that exposure to high-concentration SiNPs activates the pro-apoptotic pathway mediated by caspase-3 and inflammatory and oxidative stress pathway by high expression of iNOS by immunohistochemical findings. Testicular productions of ROS with impairment of fertility and activation of the antioxidant defense system have been reported after exposure to toxic substances [26]. ROS also enhance the oxidation of vital compounds such as lipid, where membranes of sperms are rich in unsaturated fatty acids which make them susceptible to oxidative damage, then apoptosis [27]. Excessive ROS has been shown to cause oxidative damage to the plasma membrane, which leads to impaired sperm function [28]. In addition, the overproduction of ROS is considered to be potentially dangerous to sperms due to oxidation of polyunsaturated fatty acids in lipids, amino acids, and protein; induction of DNA break; and subsequent apoptosis [29].

## Conclusion

The present study proved that SiNPs in high concentrations might exert a toxic effect on the male reproductive system; this mechanism could be related to DNA damage, inflammation, oxidative stress, and apoptosis.

## Data Availability

The datasets generated during and/or analyzed during the current study are available on reasonable request.
